# Sex-disaggregated effectiveness data reporting in COVID-19 vaccine research: a systematic review

**DOI:** 10.1038/s43856-023-00297-7

**Published:** 2023-05-19

**Authors:** Giorgia Sulis, Ji Yoon Kim, Valérie Rodrigue, Geneviève Gore, Alexandra Peebles, Angela K. Ulrich, Miranda Horn, Nicole E. Basta

**Affiliations:** 1 School of Epidemiology and Public Health, Faculty of Medicine, University of Ottawa, Ottawa, ON Canada; 2 Clinical Epidemiology Program, Ottawa Hospital Research Institute, Ottawa, ON Canada; 3 Department of Epidemiology, Biostatistics and Occupational Health, School of Population and Global Health, Faculty of Medicine and Health Sciences, McGill University, Montreal, QC Canada; 4 Schulich Library of Physical Sciences, Life Sciences, and Engineering, McGill University, Montreal, QC Canada; 5 Center for Infectious Disease Research and Policy, University of Minnesota, Minneapolis, MN USA

**Keywords:** Epidemiology, Viral infection

## Abstract

**Background:**

Sex and gender are believed to influence vaccine response. Yet, the relationship between sex and gender and COVID-19 vaccine efficacy is poorly understood and remains under-investigated.

**Methods:**

We conducted a systematic review to determine whether and to what extent post-approval COVID-19 vaccine effectiveness (VE) studies report sex-disaggregated VE data.

We searched four publication and pre-publication databases and additional grey literature sources for relevant published/preprint studies released between 1 January 2020 and 1 October 2021 (i.e., pre-Omicron era). We included observational studies providing VE estimates for one or more licensed/approved COVID-19 vaccines and including both males and females. Two reviewers independently assessed study eligibility, extracted data, and assessed risk-of-bias through a modified version of Cochrane’s ROBINS-I tool. A qualitative data synthesis was performed.

**Results:**

Here we show that, among 240 eligible publications, 68 (28.3%) do not report the sex distribution among participants. Only 21/240 (8.8%) studies provide sex-disaggregated VE estimates, and high between-study heterogeneity regarding design, target population, outcomes, and vaccine type/timing prevent the assessment of sex in determining COVID-19 VE across studies.

**Conclusions:**

Our findings indicate that few COVID-19 vaccine research publications account for sex. Improved adherence to recommended reporting guidelines will ensure that the evidence generated can be used to better understand the relationship between sex and gender and VE.

## Plain Language Summary

The level of protection that vaccines provide against COVID-19 might depend on a person’s sex or gender. However, sex and gender are not always reported in studies on the effectiveness of COVID-19 vaccines. Here, we systematically reviewed the literature on COVID-19 vaccine effectiveness and looked at whether the studies we found separated out their data on vaccine effectiveness by participants’ sex. Out of the 240 publications we identified, 68 (28.3%) did not report the sex of the participants in their study, and only 21 studies (8.8%) reported vaccine effectiveness data separated by sex. These results show that a substantial proportion of COVID-19 vaccine research publications do not account for sex. Efforts should be made by researchers to study and report the relationship between sex and vaccine effectiveness, to help to optimise vaccination strategies so that all people are adequately protected.

## Abstract (web summary)

Sulis et al. perform a systematic review to investigate to what extent post-approval COVID-19 vaccine effectiveness studies report sex-disaggregated data. They identified 240 relevant publications, of which only a small percentage (8.8%) provided sex-disaggregated effectiveness estimates.

## Introduction

The importance of considering sex as a biological variable (SABV) and gender as a social construct in statistical analyses is receiving increasing recognition^1–4^. In recent decades, funding agencies and regulatory authorities have introduced requirements to include both male and female participants and to report sex-disaggregated results in biological research, focusing primarily on preclinical studies and clinical trials^5^. In 2016, the Gender Policy Committee of European Association of Science Editors developed the Sex and Gender Equity in Research (SAGER) guidelines^6^, which have been widely adopted by scientific publishers to encourage authors to report sex- and gender-specific results more systematically and transparently across study types.

A growing body of evidence indicates that sex and gender are associated with and are among key factors in shaping immunogenicity, pharmacokinetics, and vaccine response^7–9^. In particular, sex differences in reported adverse events following vaccination is one of the clearest examples that we have of the role of sex in vaccine response^10,11^. Furthermore, multiple factors, ranging from the recipient and pathogen-related characteristics to context-specific aspects, can affect a vaccine’s ability to induce an immune response and confer protection against a range of key outcomes, but these are often underexplored in preclinical and clinical trials^12^. The scope and scale of COVID-19 vaccine research published since 2020 provides an important opportunity to assess the degree to which sex is being considered in vaccine effectiveness (VE) studies. While reduced VE for many vaccines among older age groups due to immunosenescence is well-recognized^13^, the degree to which sex and age-sex interaction impact VE is less clear and less frequently investigated and reported. In fact, females typically present stronger immune responses, which might lead to greater VE compared to males^14,15^. The relationship between sex and immune response to vaccination may therefore have important implications for COVID-19 prevention, especially given that male sex has been identified as a risk factor for severe COVID-19 and COVID-19-related death^16^.

Hundreds of vaccine clinical trials have been conducted since the start of the COVID-19 pandemic, and an estimated 50 COVID-19 vaccines have been approved in at least one country as of 2 December 2022^17^. Yet, a systematic review of COVID-19 vaccine clinical trials that were published by 22 April 2021 found that only 24% of the included trials reported sex-disaggregated estimates for their primary outcome^18^. Considering the limited availability of sex-disaggregated efficacy data from COVID-19 vaccine clinical trials^18^, post-approval observational studies investigating VE offers an opportunity to provide additional sex-specific estimates relevant to vaccine evaluation and can help advance our understanding of the relationship between sex and vaccine response. In this study, we examine the literature for observational COVID-19 VE studies to determine the proportion of studies that reported vaccine effectiveness disaggregated by sex and compare the characteristics of these studies. After collating the evidence from eligible COVID-19 VE studies identified through systematic searches, we show that a substantial proportion of studies did not report participants’ sex and/or failed to account for sex in their analyses. Furthermore, we find that a very small number of studies reported sex-disaggregated VE data, thus missing an important opportunity to shed light on whether and how sex affects a person’s response to COVID-19 vaccines.

## Methods

We conducted a systematic review of the published literature based on a prespecified protocol that was registered in the International Prospective Register of Systematic Reviews (PROSPERO; identifier: CRD42021289263). We followed the Preferred Reporting Items for Systematic Reviews and Meta-analyses (PRISMA) guidelines 2020, as indicated in the PRISMA checklists^19,20^.

### Search strategy

Using combinations of terms related to the concepts of “COVID-19 vaccine” and “effectiveness”, we searched Ovid MEDLINE, Europe PMC Preprints, Embase (Ovid) and the WHO COVID-19 Research Database for relevant published or preprint studies released between 1 January 2020 and 1 October 2021 (Supplementary Table 1). We retrieved additional records through hand-searching websites of relevant research initiatives and public health agencies such as the COVID-19 Evidence Network to support Decision-Making (COVID-END), the International Vaccine Access Center of the Johns Hopkins University, the UK Health Security Agency, and the Public Health Agency of Canada (PHAC) as of 27 October 2021. Where available, we applied filters to exclude studies based on animal models; no restrictions were placed with respect to the language of publication. For all eligible preprints based on inclusion and exclusion criteria as detailed below, we searched for any corresponding published version of the study up to 15 September 2022 and used the published version for data extraction. Fully reproducible search strategies are available in Supplementary Note 1.

### Review process

Two reviewers among GS, VR, JK, AP, MH, and AU independently screened each record first by title and abstract, and then in full text based on predefined eligibility criteria. Data extraction and risk of bias assessment of eligible studies were also carried out independently by two reviewers among those listed above. At all steps, disagreements were solved by discussion or arbitration of a third author (GS).

### Study eligibility criteria

All observational studies that reported on the effectiveness of one or more COVID-19 vaccines and their combinations for those COVID-19 vaccines that had been approved/authorized for use were eligible. The list of eligible vaccines was obtained from the list of approved/authorized vaccines reported in the COVID-19 Vaccine Development and Approvals Tracker (covid19.trackvaccines.org) at the time of the search^17^. Studies were included if they met the eligibility criteria regardless of the type of measure being reported and the outcome event(s) of interest (e.g., documented test positivity, symptomatic infection, severe disease, hospitalization, infection transmission etc.). No restrictions were placed on the geographic area where the studies were conducted.

We excluded non-primary studies (e.g. reviews, commentaries, opinion pieces), study protocols without reporting of results, studies without a control group (e.g. case series, studies including only vaccinated individuals), clinical trials (as these studies estimate vaccine efficacy but not effectiveness), economic analyses, modelling studies, qualitative studies, and studies that did not specify the type of vaccine(s) under investigation.

### Risk of bias assessment

We assessed the risk of bias in all studies that reported sex-specific VE data using Cochrane’s ROBINS-I (Risk Of Bias In Non-randomized Studies of Interventions) tool^21^, adapted to our research question as detailed in Supplementary Note 2.

### Data extraction and synthesis

For each COVID-19 VE study that met the eligibility criteria described above, we recorded whether it reported sex-disaggregated VE data. For studies not reporting such data, we included only the following details: bibliographic information, type of publication (published refereed article, preprint, report from a public health agency), study country, vaccine(s) under evaluation, type of study population, whether the sex distribution of the study population was reported, and percentage of female participants included in the study. For the purpose of this review, study participants were considered exposed if they had received at least one dose of any COVID-19 vaccine that had been approved, authorized, licensed, granted emergency use authorization, or made available for use outside of clinical trials via any pathway by a regulatory agency, a national authority, or another entity.

For studies that reported sex-disaggregated VE data, we developed a standardized data extraction form that was pilot tested by all members of the review team on five randomly selected studies and refined as needed. Through this form, we collected the same basic details as indicated above for studies not reporting sex-disaggregated VE data, along with additional study-level data including (but not limited to): study period, study methodology, setting, primary variant circulating, population characteristics (e.g. demographics, prior infection status), vaccine type and schedule, the timing of outcome assessment relative to dose administration, number of vaccinated and unvaccinated individuals with and without the events of interest, VE estimates and other relevant outcomes.

For all studies, country groupings were based on the geographic region classification of the United Nations Department of Economic and Social Affairs and the income classification of the World Bank^22,23^.

Meta-analyses of sex-specific VE were planned but could not be undertaken owing to the high between-study heterogeneity in terms of populations, vaccine types, outcomes, and approaches. Therefore, we only performed a qualitative synthesis of the evidence.

### Reporting summary

Further information on research design is available in the Nature Portfolio Reporting Summary linked to this article.

## Results

**Fig. 1:**
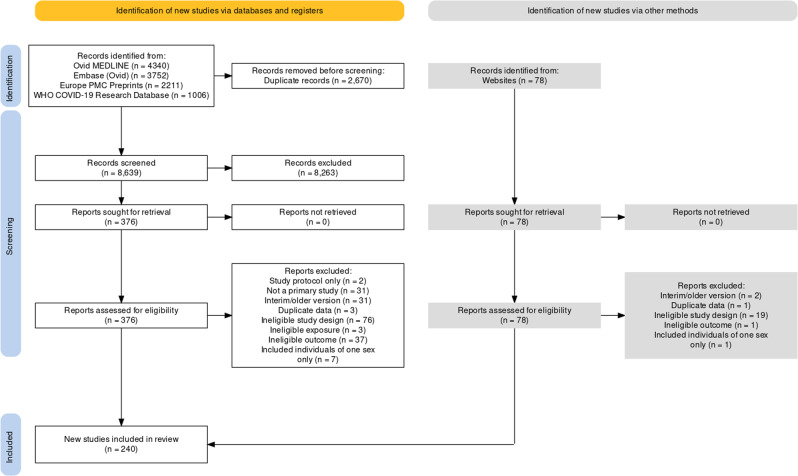
PRISMA flow diagram. The diagram details the search and selection process applied in the review.

After screening 8639 unique records, we identified 376 studies eligible for full-text review and 78 additional records retrieved through other sources (Fig. 1). We determined that 248 studies of COVID-19 VE met the eligibility criteria. Of these, eight studies included participants of one sex only—five were studies on pregnant women, two were carried out among incarcerated males, and one involved an all-male population of workers within the Indian Armed Forces. The list of studies excluded after full-text review and their reasons for exclusion are provided in Supplementary Data 1.

The main features of the 240 studies that included both males and females in the study population are summarized in Table 1 and full references are provided in Supplementary Data 2. While most studies accounted for sex in their analyses (e.g., by matching on sex or adjusting for sex in regression models), 68/240 (28.3%) studies did not explicitly report the sex distribution of their study population. Of these, the vast majority (54/68, 77.9%) were published in peer-reviewed journals, 12 (17.7%) were preprints, and 3 (4.4%) were reports from public health agencies. Across all other studies, the percentage of female individuals ranged from 2.9 to 95.4% (median, IQR: 54.2%, 49.0–63.9) (Table 1), reflecting the demographic characteristics of the population under assessment (e.g., fewer male participants are typically enrolled in studies of healthcare workers because most individuals in this occupational group are female) and/or selection bias issues (e.g., females less likely than males to be recruited into the study in some contexts).

**Table 1:** Main features of 240 studies investigating COVID-19 vaccine effectiveness that met the criteria for inclusion in this review.

Characteristic	COVID-19 VE studies		
	All (*N* = 240) *n* (%)	Studies reporting sex-disaggregated VE (*N* = 21) *n* (%)	Studies not reporting sex-disaggregated VE (*N* = 219) *n* (%)
Publication type			
Published refereed article	207 (86.3)	19 (90.5)	188 (85.8)
Preprint article	30 (12.5)	2 (9.5)	28 (12.8)
Report from a public health agency	3 (1.2)	0 (0)	3 (1.4)
Study country income level			
High income	205 (85.4)	17 (81.0)	188 (85.8)
Low or middle income	35 (14.6)	4 (19.0)	31 (14.2)
Study country geographic area			
Asia	59 (24.6)	5 (23.8)	54 (24.7)
Europe	87 (36.3)	6 (28.6)	81 (37.0)
Latin America and the Caribbean	15 (6.3)	2 (9.5)	13 (5.9)
Northern America	79 (32.9)	8 (38.1)	71 (32.4)
Population group			
General population	116 (48.3)	15 (71.4)	101 (46.1)
Older adults	20 (8.3)	2 (9.5)	18 (8.2)
Healthcare workers	53 (22.1)	2 (9.5)	51 (23.3)
US Veterans	8 (3.3)	2 (9.5)	6 (2.7)
Children/Adolescents	3 (1.3)	0 (0)	3 (1.4)
Other special population*	40 (16.7)	0 (0)	40 (18.3)
Vaccine products under investigation			
Any mRNA vaccine	60 (25.0)	5 (23.8)	55 (25.1)
Comirnaty (Pfizer/BioNTech)	65 (27.1)	5 (23.8)	60 (27.4)
Spikevax (Moderna)	5 (2.1)	1 (4.8)	4 (1.8)
Vaxzevria (Oxford/AstraZeneca) and/or Covishield (Serum Institute of India)	11 (4.6)	2 (9.5)	9 (4.1)
Jcovden (Janssen)	3 (1.3)	0 (0)	3 (1.4)
Sputnik V (Gamaleya)	3 (1.3)	2 (9.5)	1 (0.5)
Coronavac (Sinovac) and/or other inactivated vaccines	5 (2.1)	1 (4.8)	4 (1.8)
Any mRNA vaccines and viral vector-based vaccines	72 (30.0)	5 (23.8)	67 (30.6)
Any viral-vector-based vaccine and any inactivated vaccine	9 (3.8)	0 (0)	9 (4.1)
Other	7 (2.9)	0 (0)	7 (3.2)
Sex distribution of study population			
Reported	172 (71.7)	21 (100)	151 (68.9)
Not reported	68 (28.3)	0 (0)	68 (31.1)
Percentage of female participants (if available)			
Median (IQR)	54.0 (49.0–63.9)	52.4 (50.9–57.3)	54.5 (48.5–65.7)
Range	2.9–95.4	9.5–84.0	2.9–95.4

^*^ This category includes a range of groups such as individuals residing in long-term care facilities, incarcerated people, employees in a particular firm/sector, and individuals with a specific medical condition (e.g. dialysis patients, transplant recipients, people who underwent surgery, etc).

*IQR* interquartile range, *VE* vaccine effectiveness.

Only 21/240 (8.8%) studies provided sex-specific VE estimates or provided sufficient aggregated data allowing to calculate VE for one or both sexes. Across the 21 studies that reported COVID-19 VE data disaggregated by sex, we found substantial heterogeneity with respect to the design and methods of data collection, the study populations, the vaccine(s) under investigation, the types of outcome events against which effectiveness was assessed, and the timing of VE evaluation relative to the last vaccine dose (Supplementary Data 3). For all studies reporting sex-specific VE data, the risk of bias was judged to be moderate or serious mostly due to the high potential for confounding along with moderate concerns regarding selection bias and outcome misclassification (Supplementary Data 4).

We did not observe meaningful differences with respect to the country income level, the geographic areas where the study was carried out, or the vaccine(s) under investigation between studies that reported sex-disaggregated measures of COVID-19 VE versus those that did not (Table 1). Most studies reporting VE by sex were conducted in the general population (15/21, 71.4%). Among the 219 studies not reporting sex-specific VE data, almost half (101, 46.1%) involved the general population, 51 (23.3%) were restricted to healthcare workers, 18 (8.2%) focused on older adults, and 40 (18.3%) on other special populations (e.g., children/adolescents, residents of long-term care facilities, employees of a particular sector/firm, etc.).

## Discussion

The importance of evaluating the role of sex and gender across a range of biological processes has become more widely recognized by the scientific community and research funding agencies in recent years, and many publishing and reporting guidelines have devoted efforts to standardize reporting. Nonetheless, opportunities for improvement remain in evaluating the role of sex and gender in vaccine research. In this systematic review, we found that less than 10% of eligible post-vaccine authorization/approval observational studies investigating the effectiveness of one or more COVID-19 vaccines reported sex-disaggregated VE estimates. Therefore, we are missing an opportunity to investigate thoroughly the role of sex in determining an individual’s response to COVID-19 vaccines.

Almost all studies identified in our review included male and female participants in variable proportions, but—among those that considered sex in their analyses—sex was mainly handled as a potential confounder. As such, it was often used for matching and/or included in multivariable regression models aimed at estimating adjusted VE. However, to elucidate whether males and females respond differently to vaccination, sex should be investigated as a potential effect modifier. To this end, sex-specific VE estimates are needed^24^. Given that only a highly heterogenous group of 21 studies did provide such sex-specific estimates, whether sex plays a clinically relevant role in determining COVID-19 VE remains unclear.

Our work was focused on the variable sex, which must be clearly differentiated from gender, though both these variables need to be investigated further. The complex set of socially constructed roles, behaviours, and identities that define an individual’s gender is worth noting, as this is also widely neglected as a potential factor shaping effectiveness estimates. Without systematic and transparent reporting of sex- and gender-stratified analyses, as well as adjusted estimates, we simply lack the necessary information to understand whether and how these important factors contribute to determining the effectiveness of any COVID-19 vaccine. Gender may be just as likely, or more likely, than biological sex to confound the relationship between vaccination and vaccine effectiveness, by affecting both the exposure (whether someone gets vaccinated) and the outcome (whether the vaccine succeeds at preventing a given event, e.g., severe COVID-19). Notably, research has shown that both sex and gender are associated with factors that directly influence the outcome such as immunological responses to immunization and the likelihood of acquiring an infection^25,26^, and can play a role in determining an individual’s access to vaccination and their willingness to get vaccinated^27,28^.

Of note, over 85% of the studies included in our review were articles published in peer-reviewed journals (as opposed to preprints or reports from public health agencies), suggesting that the principles outlined in the SAGER guidelines^6^, which have already been endorsed by several journals, are not systematically applied, if incorporated at all, within a journal’s publication policies. Therefore, greater efforts should be made to increase researchers’ awareness of, and adherence to, recommended and/or required reporting practices, especially given that these practices are beneficial to not only the scientific community, but society at large, in addition to being relatively easy to implement. In fact, understanding whether vaccine effectiveness varies by sex and gender has important implications for vaccination policies and practices as this could lead to sex- and/or gender-specific recommendations regarding dosages, intervals between doses, or preferred vaccines, in order to maximize the benefits of vaccination. Failing to report sex- and gender-disaggregated VE estimates hampers the ability to draw conclusions on whether relevant differences exist between sexes and genders with respect to COVID-19 vaccine effectiveness. Instead, systematic reporting of sex- and gender-disaggregated estimates, regardless of statistical significance, would not only clarify the role (or lack thereof) of sex and gender in vaccine research but also avoid publication bias.

The biggest limitation of this systematic review is that, given the timing of the search, we only included studies conducted and released during the pre-Omicron era. It is to be noted that as our search strategy was limited to research conducted and released up to October 2021, our systematic review also does not capture all relevant studies carried out right before the emergence of Omicron as some were released later. While it is possible that a more substantial proportion of studies published after October 2021 reported sex-disaggregated VE data compared to previous studies, major changes in reporting practices occurring abruptly over the past year are unlikely. As such, we believe that our findings apply more broadly and highlight key concerns about reporting practices.

Our findings call for renewed attention to the widespread lack of reporting of results relevant to the role of sex and gender in COVID-19 vaccine effectiveness observational studies and the need for a substantial paradigm change. Simple, yet highly impactful, steps can be taken to standardize and normalize this practice in the research community. For example, accounting for sex and gender can be made mandatory for any manuscript submitted for publication, similar to ethics statements or funding disclosure requirements. Our review provides solid evidence that additional efforts are needed urgently to improve reporting of sex-related differences in responses to vaccines, including but not limited to those aimed at preventing COVID-19 and its complications.

## Supplementary information

Supplementary Information

Supplementary Data 1

Supplementary Data 2

Supplementary Data 3

Supplementary Data 4

Description of Additional Supplementary Files

Reporting Summary

## Data Availability

Our review protocol is publicly available in the International Prospective Register of Systematic Reviews (PROSPERO; identifier: CRD42021289263), and our fully reproducible search strategies are provided in the supplementary material. Template data collection forms can be obtained from the corresponding author upon reasonably motivated request. Data extracted from included studies are reported in the article and its supplementary material along with references of all studies included and excluded after full-text review.
