# Supplementary material for: Sex-disaggregated effectiveness data reporting in COVID-19 vaccine research: a systematic review
Source: Commun Med (Lond). 2023 May 19;3:69. doi: 10.1038/s43856-023-00297-7 (PMC10196298; doi:10.1038/s43856-023-00297-7)
Supplement: Supplementary file 1 — Supplementary Information [file 43856_2023_297_MOESM1_ESM.pdf]

Supplementary Table 1: Search strategy overview

Platform	Database(s)	Database coverage dates	# Results	Search Date	Remarks
OvidSP	Ovid MEDLINE ALL(R)	1946 -	4340	2021/10/01	Excluded studies indexed as animal only or with animal terms in title or clinical trials (I, II, III) or RCTs
OvidSP	EMBASE	1996 -	3752	2021/10/01	Excluded studies indexed as animal only or with animal terms in title or clinical trials (I, II, III) or RCTs or conference materials
Europe PMC	Preprints		2211	2021/10/01	Restricted search; truncation not supported; includes MedRxiv and other preprints: https://europepmc.org/Preprints
WHO	COVID-19 Database: Global Literature/Research on Coronavirus Disease		1006	2021/10/01	Excluded records from MEDLINE, Embase, medRxiv, bioRxiv, ICTRP
			10303		
	**TOTAL NUMBER OF RECORDS**		11309		

Supplementary Note 1: Search strategies

Limits or filters used: 2020-

Number of records identified through database searching: 11309

Original searches:

Ovid MEDLINE(R) ALL <1946 to September 30, 2021> (Ovid)

1 (ZF2001 or RBD-Dimer or Zifivax or Covaxin or BBV152* or Ad5-nCoV or Convidecia or CIGB-66 or Abdala or KoviVac or EpiVacCorona or Epi-Vac-Corona or "Sputnik Light" or "Sputnik V" or Gam-COVID-Vac or "Ad26.COV2.S" or Ad26COV2S or Ad26COVS1 or JNJ-78436735 or QazVac or QazCovid-in or MVC-COV1901 or "SARS-CoV-2 Vaccine" or KCONVAC or mRNA-1273 or Spikevax or AZD1222 or ChAdOx1* or Vaxzevria or BNT162* or Tozinameran or Comirnaty or Covishield or "COVID-19 Inactivated Vaccine" or COVIran Barekat or BBIBP-CorV or CoronaVac or TAK-919 or ZyCoV-D).mp. 2238

2 (Abdavomeran or ad5ncov or bnt 162 or chadox 1 or bbv 152* or gam-kovid-vak or rAd26* or rAd5-s or ganulameran or bnt 162b* or "cx 024414" or cx024414 or messenger RNA-1273 or messenger RNA1273 or mRNA1273 or tak919 or tozinameran or comirnaty or "pf 07302048" or pf07302048).mp. 231

3 ((exp vaccines/ or exp vaccinations/ or immunization/ or immunization schedule/ or immunization, secondary/ or exp immunotherapy, active/ or immunization programs/) and (COVID-19/ or SARS-CoV-2/)) or COVID-19 vaccines/ 6555

4 ((covid or covid19 or ncov or SARS-COV-2 or SARS-COV2 or SARSCOV-2 or SARSCOV2 or coronavirus) and (Anhui Zhifei Longcom or Bharat Biotech or CanSino or "Center for Genetic Engineering and Biotechnology" or CIGB or Chumakov Center or FBRI or Gamaleya or Janssen or "Johnson & Johnson" or Kazakhstan RIBSP or Medigen or Minhai Biotechnology Co or Moderna or AstraZeneca or Pfizer or "Serum Institute of India" or "Shifa Pharmed Industrial Co" or Sinopharm or Sinovac or Takeda or Zydus Cadila) and (vaccin$ or immuniz$ or immunis$)).ti,ab,kf. 1060

5 ((covid or covid19 or ncov or SARS-COV-2 or SARS-COV2 or SARSCOV-2 or SARSCOV2 or coronavirus) and (vaccin$ or immuniz$ or immunis$)).ti,kf. 10215

6 1 or 2 or 3 or 4 or 5 12496

7 epidemiologic studies/ or exp case-control studies/ or exp cohort studies/ or controlled before-after studies/ or cross-sectional studies/ or historically controlled study/ or interrupted time series analysis/ 2761088

8 (case control or cohort or follow up or followup or incidence or longitudinal* or matched or odds ratio* or observational* or prevalence or prospective* or retrospective* or risk ratio* or "before after" or "before and after" or "controlled study" or "cross sectional*" or "interrupted time series" or "real world" or "repeated measures").ti,ab,kf. 4790346

9 (effectiveness or efficacy or (post adj3 (implementation or vaccin*)) or postimplementation or postvaccin*).ti,ab,kf. 1349827

10 (assess* or compar* or effect* or evaluat* or impact* or prevent* or protect*).ti. 4295257

11 7 or 8 or 9 or 10 9458131

12 6 and 11 4749

13 (exp animals/ not human*.sh.) or (animal* or bat or bats or cat or cats or chicken* or dog or dogs or duck or ducks or in vitro or pig or pigs or preclinical or pre clinical or ferret* or hamster* or macaque* or mink or monkey* or mouse or mice or primate* or rat or rats).ti. or (randomized controlled trial or clinical trial, phase i or clinical trial, phase ii or clinical trial, phase iii or clinical trial, veterinary or clinical trials, veterinary as topic or clinical trial protocol or clinical trial protocols as topic).pt. 6044289

14 12 not 13 4393

15 limit 14 to yr="2020 -Current" 4340

Embase <1996 to 2021 Week 38> (Ovid)

1 exp SARS-CoV-2 vaccine/ 6454

2 (ZF2001 or RBD-Dimer or Zifivax or Covaxin or BBV152* or Ad5-nCoV or Convidecia or CIGB-66 or Abdala or KoviVac or EpiVacCorona or Epi-Vac-Corona or "Sputnik Light" or "Sputnik V" or Gam-COVID-Vac or "Ad26.COV2.S" or Ad26COV2S or Ad26COVS1 or JNJ-78436735 or QazVac or QazCovid-in or MVC-COV1901 or "SARS-CoV-2 Vaccine" or KCONVAC or mRNA-1273 or Spikevax or AZD1222 or ChAdOx1* or Vaxzevria or BNT162* or Tozinameran or Comirnaty or Covishield or "COVID-19 Inactivated Vaccine" or COVIran Barekat or BBIBP-CorV or CoronaVac or TAK-919 or ZyCoV-D).mp. 6950

3 (Abdavomeran or ad5ncov or bnt 162 or chadox 1 or bbv 152* or gam-kovid-vak or rAd26* or rAd5-s or ganulameran or bnt 162b* or "cx 024414" or cx024414 or messenger RNA-1273 or messenger RNA1273 or mRNA1273 or tak919 or tozinameran or comirnaty or "pf 07302048" or pf07302048).mp. 1195
4 ((covid or covid19 or ncov or SARS-COV-2 or SARS-COV2 or SARSCOV-2 or SARSCOV2 or coronavirus) and (Anhui Zhifei Longcom or Bharat Biotech or CanSino or "Center for Genetic Engineering and Biotechnology" or CIGB or Chumakov Center or FBRI or Gamaleya or Janssen or "Johnson & Johnson" or Kazakhstan RIBSP or Medigen or Minhai Biotechnology Co or Moderna or AstraZeneca or Pfizer or "Serum Institute of India" or "Shifa Pharmed Industrial Co" or Sinopharm or Sinovac or Takeda or Zydus Cadila) and (vaccin$ or immuniz$ or immunis$)).ti,ab,kf. 919
5 ((covid or covid19 or ncov or SARS-COV-2 or SARS-COV2 or SARSCOV-2 or SARSCOV2 or coronavirus) and (vaccin$ or immuniz$ or immunis$)).ti,kf. 9356
6 1 or 2 or 3 or 4 or 5 11591
7 exp case control study/ or cohort analysis/ or cross-sectional study/ or follow up/ or incidence/ or prevalence/ or prospective study/ or retrospective study/ 4565815
8 (case control or cohort or follow up or followup or incidence or longitudinal* or matched or odds ratio* or observational* or prevalence or prospective* or retrospective* or risk ratio* or "before after" or "before and after" or "controlled study" or "cross sectional*" or "interrupted time series" or "real world" or "repeated measures").ti,ab,kf. 6369650
9 (effectiveness or efficacy or (post adj3 (implementation or vaccin*)) or postimplementation or postvaccin*).ti,ab,kf. 1751542
10 (assess* or compar* or effect* or evaluat* or impact* or prevent* or protect*).ti. 4021416
11 7 or 8 or 9 or 10 10641110
12 6 and 11 4484
13 limit 12 to (randomized controlled trial or phase 1 clinical trial or phase 2 clinical trial or phase 3 clinical trial) 147
14 12 not 13 4337
15 (conference abstract or conference paper or "conference review").pt. 4720315
16 (exp animal/ or animal experiment/ or nonhuman/) not (exp human/ or human experiment/) 4604334
17 (animal* or bat or bats or cat or cats or chicken* or dog or dogs or duck or ducks or in vitro or pig or pigs or preclinical or pre clinical or ferret* or hamster* or macaque* or mink or monkey* or mouse or mice or primate* or rat or rats).ti. 1574911
18 14 not (15 or 16 or 17) 3786
19 limit 18 to yr="2020 -Current" 3752

Europe PMC: Preprints

TITLE:((covid OR covid19 OR ncov OR SARS-COV-2 OR SARS-COV2 OR SARSCOV-2 OR SARSCOV2 OR coronavirus) AND (ZF2001 OR Covaxin OR Ad5-nCoV OR CIGB-66 OR KoviVac OR EpiVacCorona OR "Sputnik Light" OR "Sputnik V" OR "Ad26.COV2.S" OR QazVac OR MVC-COV1901 OR mRNA-1273 OR AZD1222 OR BNT162b2 OR Covishield OR BBIBP-CorV OR Sinopharm OR CoronaVac OR TAK-919 OR ZyCoV-D OR vaccinated OR vaccination OR vaccinations OR vaccine OR vaccines OR immunization OR immunizations OR immunized OR immunisation OR immunisations OR immunised)) AND (("case control" OR cohort OR "follow up" OR followup OR incidence OR longitudinal OR longitudinally OR matched OR "odds ratio" OR "odds ratios" OR observational OR prevalence OR prospective OR retrospective OR "risk ratio" OR "risk ratios" OR "before after" OR "before and after" OR "controlled study" OR "cross sectional" OR "interrupted time series" OR "real world" OR "repeated measures" OR effectiveness OR efficacy OR "post implementation" OR "post vaccination" OR postimplementation OR postvaccination) OR TITLE:(assess OR assessing OR assessment OR compare OR comparing OR comparison OR effect OR effects OR evaluate OR evaluating OR evaluation OR impact OR prevent OR preventing OR prevention OR protect OR protecting OR protection)) AND (SRC:PPR)
2211 records on October 1, 2021

WHO COVID-19 Database: Global Literature/Research on Coronavirus Disease

https://search.bvsalud.org/global-literature-on-novel-coronavirus-2019-ncov/?lang=en
(
ti:(ZF2001 OR Covaxin OR Ad5-nCoV OR CIGB-66 OR KoviVac OR EpiVacCorona OR Epi-Vac-Corona OR "Sputnik Light" OR "Sputnik V" OR "Ad26.COV2.S" OR QazVac OR MVC-COV1901 OR mRNA-1273 OR AZD1222 OR BNT162b2 OR Covishield OR BBIBP-CorV OR Sinopharm OR CoronaVac OR TAK-919 OR ZyCoV-D OR vaccin* OR immuniz* OR immunis*)
OR

su:(ZF2001 OR Covaxin OR Ad5-nCoV OR CIGB-66 OR KoviVac OR EpiVacCorona OR Epi-Vac-Corona OR "Sputnik Light" OR "Sputnik V" OR "Ad26.COV2.S" OR QazVac OR MVC-COV1901 OR mRNA-1273 OR AZD1222 OR BNT162b2 OR Covishield OR BBIBP-CorV OR Sinopharm OR CoronaVac OR TAK-919 OR ZyCoV-D OR vaccin* OR immuniz* OR immunis*)
)
AND
(
tw:("case control" OR cohort OR "follow up" OR followup OR incidence OR longitudinal* OR matched OR "odds ratio*" OR observational OR prevalence OR prospective* OR retrospective* OR "risk ratio*" OR "before after" OR "before and after" OR "controlled study" OR "cross sectional" OR "interrupted time series" OR "real world" OR "repeated measures" OR effectiveness OR efficacy OR "post implementation" OR "post vaccin*" OR postimplementation OR postvaccin*)
OR
ti:(assess* OR compar* OR effect* OR evaluat* OR impact OR prevent* OR protect*)
)
Excluded records from MEDLINE, Embase, medRxiv, bioRxiv, ICTRP
1006 records on October 1, 2021

Supplementary Note 2: Risk of bias assessment methods

The risk-of-bias assessment tool utilized in this systematic review is based on Cochrane's ROBINS-I. Two of the seven domains that are included in ROBINS-I (bias due to deviations from intended interventions and bias in selection of the reported result) were deemed less relevant to our research question and were thus excluded. The following domains are covered:

1) Bias due to confounding
2) Bias in participants' selection
3) Bias in classification of interventions
4) Bias due to missing data
5) Bias due to outcome misclassification

Key bias sources and suggested judgement of potential scenarios were defined in line with what done in COVID-END assessments.

Domain 1: Bias due to confounding

- **Sources of confounding to be considered** include:
  - accounting for non-immune period (i.e. first 14 days post vaccination),
  - inclusion of individuals with prior SARS-CoV-2 infection,
  - accounting for calendar time (risk of exposure and access to vaccination may vary over time),
  - adjustment for factors that affect both access/response to vaccination and infection risk/disease severity (e.g. age, race, socioeconomic factors, occupation, underlying chronic conditions, etc.).

- **Possible scenarios and related judgement:**
  - If non-immune period not accounted for:
    - Presence of an effect during non-immune period (i.e. vaccine results to be effective when VE measured <14 days post dose 1) or result not reported → **Moderate risk**
    - Unclear whether the non-immune period was taken into account (e.g. timing of VE measurement not specified) → **Serious risk**
  - If the study population includes a mix of individuals with and without prior infection:
    - inclusion of prior infection status as a covariate in the models → **Moderate risk**
    - No adjustment/separate analysis for previously infected vs others → **Serious risk**
  - Accounting for calendar time (not a major issue if the study observation period was short, as was the case in many early studies):
    - Use of time-varying statistics without explicit mention of adjustment for calendar time → **Moderate risk**
    - Not taken into account but short time frame (e.g. 2 months or less) → **Serious risk**
    - Not taken into account and time frame >2 months → **Critical risk**

- **Questions 1.1 to 1.8** are aimed at collecting details that are relevant to confounding issues and will need to be used in judging the risk of bias due to confounding. Answer options are as follows: Yes (Y), Probably yes (PY), Probably no (PN), No (N). No information (NI). Some questions also have a "Not applicable" (NA) option.

- **Markers for a moderate to critical risk of bias:**
  - Y/PY to questions 1.1 and/or 1.6
  - PN/N to questions 1.4. 1.5, 1.7, 1.8

Domain 2: Bias in participants' selection

- **This domain is mainly about the study design**, with the test-negative study design being less prone to bias as it minimizes the differences across groups (vaccinated vs unvaccinated). Of note, heath-seeking behaviours (which affect the probability of selection into the study) may differ by vaccination status.

- **Questions 2.1 to 2.5** are aimed at collecting details that are relevant to selection bias and will need to be used in judging the risk of bias for this domain. Answer options are as follows: Yes (Y), Probably yes (PY), Probably no (PN), No (N). No information (NI). Some questions also have a "Not applicable" (NA) option.

- **Markers for a moderate to critical risk of bias:**
  - Y/PY to questions 2.1, 2.2, 2.3
  - PN/N to questions 2.4 and/or 2.5

<u>Domain 3: Bias in classification of interventions</u>

- This domain pertains to the **risk of misclassification of participants' vaccination status**, which is less likely to occur when vaccination status is determined through data linkage or consultation of vaccination registries.

- **Possible scenarios and related judgement:**
  - Source of vaccination information:
    - Database linkage study → **Low risk**
    - Questionnaire with confirmation by an additional method (e.g. registry) of at least a subset of study population → **Moderate risk**
    - Questionnaire without confirmation by an additional method → **Serious risk**
    - Estimating vaccination status based on surveillance data alone (this is the case in studies that utilize the screening method) → **Critical risk**

- **Questions 3.1 to 3.3** are aimed at collecting details that are relevant to intervention misclassification and will need to be used in judging the risk of bias for this domain. Answer options are as follows: Yes (Y), Probably yes (PY), Probably no (PN), No (N). No information (NI). Some questions also have a "Not applicable" (NA) option.

- **Markers for a moderate to critical risk of bias:**
  - PN/N to questions 3.1 and/or 3.2
  - Y/PY to question 3.3

<u>Domain 4: Bias due to missing data</u>

- This domain pertains to missing data and how the study authors handled the problem, which is likely to be a minor concern in large studies based on national registries and/or administrative datasets.

- **Questions 4.1 to 4.5** are aimed at collecting details that are relevant to missing data and will need to be used in judging the risk of bias for this domain. Answer options are as follows: Yes (Y), Probably yes (PY), Probably no (PN), No (N). No information (NI). Some questions also have a "Not applicable" (NA) option.

- **Markers for a moderate to critical risk of bias:**
  - PN/N to questions 4.1, 4.4, and/or 4.5
  - Y/PY to questions 4.2 and/or 4.3

<u>Domain 5: Bias due to outcome misclassification</u>

- **This domain pertains to the potential for misclassification of the outcome status**, including the methods and timing of outcome ascertainment and whether the approaches being used vary according to the participants' vaccination status.

- **Possible scenarios and related judgement**:
  - Testing frequency: in order to minimize bias, this has to be similar across groups, regardless of vaccination status.
    - No systematic screening but consistent methods for detection in one group vs. the other, e.g., within health networks (this is likely the case in test negative design studies unless stated otherwise) → **Moderate risk**
    - Screening performed for a subset of both study groups → **Serious risk**

        - Screening performed routinely in one study group but not in the other → **Critical risk**
    - Source of outcome data (e.g. test results, hospitalization, etc):
        - Database specifically developed to collect COVID-19-related data → **Low risk**
        - Database for non-COVID purpose but with individual level data → **Moderate risk**
        - Database for non-COVID purpose without individual level data → **Serious risk**
        - No or unclear description of database type → **Critical risk**
    - Determination of the timing of the outcome event:
        - Using a PCR positive test that was part of an ongoing standardized monitoring system (e.g., within a health network) → **Low risk**
        - Using sample date without interview or documented confirmation of symptoms within 10 days of testing (only relevant for symptomatic disease) → **Serious risk**
    - Verification of symptoms:
        - Study focused on PCR positive tests without differentiating between symptomatic and asymptomatic cases → **No information**
        - Determined based on the date of testing, with no information regarding actual symptom onset from medical records/patient reporting → **Serious risk**

- **Questions 5.1 to 5.4** are aimed at collecting details that are relevant to missing data and will need to be used in judging the risk of bias for this domain. Answer options are as follows: Yes (Y), Probably yes (PY), Probably no (PN), No (N). No information (NI). Some questions also have a "Not applicable" (NA) option.

- **Markers for a moderate to critical risk of bias:**
    - Y/PY to questions 5.1, 5.2, and/or 5.4
    - PN/N to question 5.3
