# Supplementary material for: Sex-disaggregated effectiveness data reporting in COVID-19 vaccine research: a systematic review
Source: Commun Med (Lond). 2023 May 19;3:69. doi: 10.1038/s43856-023-00297-7 (PMC10196298; doi:10.1038/s43856-023-00297-7)
Supplement: Supplementary file 6 — Description of Additional Supplementary Files [file 43856_2023_297_MOESM6_ESM.pdf]

Description of Additional Supplementary File

File Name: Supplementary Data 1

Description: List of studies excluded after full-text screening, with reasons for exclusion.

File Name: Supplementary Data 2

Description: List of included studies (n=240)

File Name: Supplementary Data 3

Description: Main characteristics of studies that reported sex-specific vaccine effectiveness (VE) estimates for one or more COVID-19 vaccines (n=21).

File Name: Supplementary Data 4

Description: Results of risk of bias assessment of 21 studies that reported sex-disaggregated vaccine effectiveness (VE) data.
